# Carotid artery dissection and non-aneurysmal subarachnoid hemorrhage following carotid endarterectomy

**DOI:** 10.1093/jscr/rjae331

**Published:** 2024-05-28

**Authors:** Lydia Kaoutzani, Klepper Alfredo Garcia, Scott Y Rahimi

**Affiliations:** Department of Neurosurgery, Wellstar Medical College of Georgia Health, 1120 15^th^ Street, Augusta, GA 30912, United States; Department of Neurology, Wellstar Medical College of Georgia Health, 1120 15^th^ Street, Augusta, GA 30912, United States; Department of Neurosurgery, Wellstar Medical College of Georgia Health, 1120 15^th^ Street, Augusta, GA 30912, United States

**Keywords:** carotid endarterectomy, carotid artery dissection, cerebral hyperperfusion syndrome

## Abstract

Stroke continues to be a major public health issue resulting in high mortality and severe long-term disability. Carotid endarterectomy (CEA) plays an important role in the prevention of ischemic stroke. Complications associated with CEA can be life threatening and prompt recognition is crucial. In this report, we present a patient who presented to the hospital with progressive headache, 2 weeks following CEA. He was neurologically intact and hypertensive. Non-contrast head computed tomography (CT) scan showed convexity subarachnoid hemorrhage (SAH). He was found to have a left internal carotid artery dissection. Patients who present to the hospital following CEA with headache and hypertension benefit from a non-contrast head CT scan. The presence of SAH can be a warning sign of cerebral hyperperfusion syndrome. Carotid artery dissection is also a disease entity that can occur in the post-operative period. Prompt recognition and treatment is crucial for the management of these disease entities.

## Introduction

Despite medical advances, stroke remains a major cause of morbidity and mortality worldwide. Carotid artery dissection is a common cause of stroke in young patients and can lead to subarachnoid hemorrhage (SAH) if the dissection occurs intracranially [1, 2]. Prevention of stroke is key in the management of this disease, and it includes medical and surgical treatment options.

Specifically, carotid endarterectomy (CEA) is recommended in patients with previous transient ischemic attacks with carotid artery stenosis between 70 and 99% and in asymptomatic patients with carotid artery stenosis of 50 and 69% [3–5]. Cerebral hyperperfusion syndrome is a rare, early post-operative complication (usually between 2 and 12 hours) following CEA [6–8]. Cerebral hyperperfusion syndrome usually presents with neurological deficits, seizures, and headaches [6–8]. The incidence of cerebral hyperperfusion syndrome has been cited to range between 0.2 and 18.9% [9, 10]. Strict blood pressure control, with medications that do not have direct effect on cerebral blood flow, appears to be the best way to improve cerebral hyperperfusion syndrome [9].

In this case study, we present a 66-year-old patient who underwent left CEA and was subsequently found to have a left carotid artery dissection and non-aneurysmal SAH.

## Case report

We describe a 66-year-old Caucasian male who was brought to the hospital for worsening left frontal and retro-orbital headache. He had undergone left CEA at an outside hospital 2 weeks prior to presentation. Patient’s past medical and surgical history were notable for hypertension, hyperlipidemia, tobacco use, right CEA and coronary artery bypass grafting surgery.

**Figure 1:**
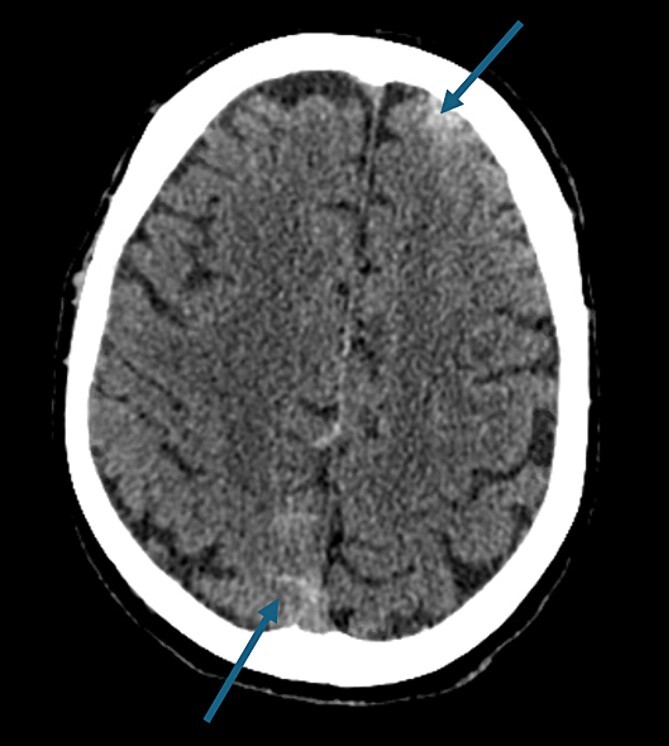
Non-contrast axial head computed tomography scan demonstrating biconvexity subarachnoid hemorrhage.

**Figure 2:**
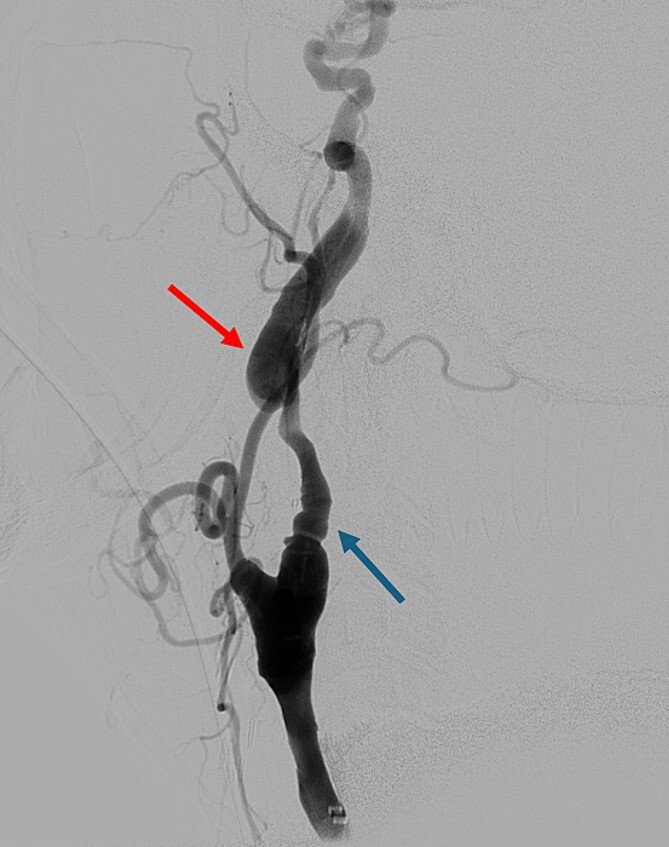
Digital subtraction angiography showing left internal carotid artery dissection (arrow on the left) with pseudoaneurysm formation (arrow on the right).

On presentation to the hospital, he was found to be in hypertensive crisis. Non-contrast head CT scan showed moderate volume biconvexity SAH (Fig. 1). CT angiography (CTA) of his head and neck showed a left internal carotid artery (ICA) dissection extending from the mid cervical segment to the level of the vertical petrous segment. Six vessel digital subtraction angiography confirmed a left ICA dissection extending from the proximal mid cervical ICA into the petrous segment. In the same region, there was also a left pseudoaneurysm formation with slight turbulent flow and stagnation (Fig. 2).

Patient underwent *Pipeline* Flex™ *embolization device* remodeling of the left carotid artery extending from the left petrous ICA to the left ICA bifurcation and anchoring of the *Pipeline* Flex™ *embolization device* on a Precise Pro Rx® Carotid Stent. To achieve this superselective catheterization of the proximal cavernous, ICA was performed with the Phenom™ 27 microcatheter. The first *Pipeline* Flex™ *embolization device* (5 × 35 mm) was deployed extending from the petrous ICA distally to the proximal petrous ICA. A second *Pipeline* Flex™ *embolization device* (5 × 35 mm) was placed overlapping the first *Pipeline* Flex™ *embolization device* and it was extending from the petrous ICA down to the distal cervical ICA. The microcatheter was advanced again over the stent to recapture the microwire. A third *Pipeline* Flex™ *embolization device* (5 × 16 mm) was deployed in the cervical ICA and telescoped in the second *Pipeline* Flex™ *embolization device*. A final stent (Precise Pro Rx® Carotid Stent 7 × 40 mm) was placed over the proximal cervical ICA irregularity extending from the previous *Pipeline* Flex™ *embolization device* down to the common carotid artery.

The patient recovered well and was discharged from the hospital. At the 2-week post-operative follow-up appointment, he was neurologically intact and non-contrast CT scan of his head showed resolution of the SAH.

## Discussion

In this paper, we present a patient who was found to have SAH and carotid artery dissection following CEA.

There have been case reports of SAH after CEAs, with no obvious carotid artery dissection, which has been attributed to cerebral hyperperfusion syndrome. In 2004, a case report of a 77-year-old female who underwent carotid artery angioplasty and stent placement for transient ischemic attack resulted in a fatal SAH [11]. The authors highlighted that elevated blood pressure after carotid artery stenting as well as severe contralateral carotid artery stenosis are risk factors that predispose to this disease [11].

In 2010, a case report of a 74-year-old male who had undergone right asymptomatic CEA presented to the emergency department on post-operative day 9 with throbbing headaches, left hemiplegia and generalized seizures [12]. He was found to have right frontal SAH with middle cerebral artery vasospasm and no identifiable cerebral aneurysm or vascular malformation [12].

In 2013, there was a case report of SAH six days following left CEA that presented with severe headache and tonic-clonic seizure, with CTA being negative for aneurysm or vascular malformation and the left ICA was patent [8]. This presentation was attributed to cerebral hyperperfusion syndrome following CEA [8].

There have been reports of intracranial carotid artery dissection resulting in SAH. Chaves et al., published a series of 10 patients with spontaneous intracranial ICA dissection [13]. Nine of these patients had strokes and one of them had SAH; the presentation of all of them was that of severe headache and contralateral hemiparesis [13]. In another case report, it was shown that cerebral carotid artery dissection secondary to a whiplash trauma resulted in fatal SAH [14].

In this case report, we present a patient who had a left sided CEA and subsequently was found to have a left carotid artery dissection and convexity SAH. We hypothesize that the carotid artery dissection could have been the result of an expanding intimal flap that can occur following CEA [15]. Subsequently, the intimal flap could have enlarged forming the dissection and pseudoaneurysm. With regards to the convexity SAH, it could had potentially been the result of the carotid artery dissection reaching the intracranial (vertical petrous) ICA segment.

## Conflict of interest statement

None declared.

## Funding

The authors did not receive any financial support from any organization for the submitted work.
